# Adipose-Derived Stem Cells (ADSC) and Aesthetic Surgery: A Mini Review

**Published:** 2013-06

**Authors:** Davood Mehrabani, Golshid Mehrabani, Shahrokh Zare, Ali Manafi

**Affiliations:** 1 Stem Cell and Transgenic Technology Research Center, Department of Pathology, Shiraz University of Medical Sciences, Shiraz, Iran; 2 Student Research Committee, School of Dentistry, International Kish Branch, Shiraz University of Medical Sciences, Shiraz, Iran; 3 Department of Plastic Surgery, Tehran University of Medical Sciences, Tehran, Iran

**Keywords:** Adipose, Stem cell, Aesthetic medicine

## Abstract

In cell therapy and regenerative medicine, a reliable source of stem cells together with cytokine growth factors and biomaterial scaffolds seem necessary. As adipose tissue is easy accessible and is abundant source of adult stem cells and can differentiate along multiple lineages, it can be considered as a good candidate in aesthetic medicine. The clinical application of adipose-derived stem cells (ASCs) is reviewed in this article.

## INTRODUCTION

Developments in stem cell science, stem cell-associated growth factors, and regenerative medicine in recent years may provide the opportunity for use of embryonic stem cells (ESCs), induced pluripotent stem cells (iPSCs), and postnatal adult stem cells in repair of tissue injuries and, eventually, in replacement of organs.^1^

Application of cellular therapy and regenerative medicine is rapidly growing, but for regenerative medicinal purposes, stem cells should meet several criteria: (i). They could be provided in abundant quantities (millions to billions of stem cells); (ii). They could be differentiated into many cell lineages in a regulatable and reproducible way; (iii). They could be harvested by minimally invasive techniques; (iv). They could safely and effectively be used in either an autologous or allogeneic host; and (v). They could be produced according to current Good Manufacturing Practice guidelines.^2^

Among stem cells, ESCs have the potential of extensive self-renewal, expansion and differentiatiation into any type of somatic tissues making them for future use in regenerative medicine.^1^ iPSCs as the other group of stem cells are derived from differentiated cells including skin fibroblasts and keratinocytes by transduction with a combination of many transcription factors involved in reprogramming. These cells are phenotypically and functionally indistinguishable from ES cells. However, their production is not dependent on a source of embryos.^3^

iPSCs could potentially be used in construction of tissue-engineered skin even several limitations were seen in practical use of iPSCs and ESCs including cellular regulation of teratoma formation, immune concerns and ethical considerations for them and problems in genetic manipulation of iPSCs.^1^^,^^4^

The third group of stem cells are postnatal adult stem cells which by their nature are immuno-compatible, and there are no ethical concerns for their use.^4^ They are called multipotent mesenchymal stem cells (MSCs) and are non-hematopoietic adult stem cells of mesodermal derivation seen in many postnatal organs and connective tissues.^4^ MSCs either derived from bone marrow or adipose tissue were shown to be suitable candidates for cell therapy. Among them, bone marrow MSCs (BM-MSCs) are a heterogeneous group of multipotent progenitor cells with the potential of self-renewal and differentiation into cells with ectodermal, mesodermal and endodermal characteristics.^5^ They have the intrinsic potential to leave the bone marrow, circulate in the blood and home to injured tissues.^6^ BM-MSCs are a standard population in regenerative medicine due to their high differentiation capacity and low morbidity during harvesting. But harvesting in bone marrow aspiration is still considered as a painful procedure and the quantity of cells acquired is usually low.^7^

MSCs, with identical characteristics to BM-MSCs were isolated from various tissues such as periosteum,^8^ trabecular bone,^9^ synovial membrane,^10^ pericytes,^11^ peripheral blood,^12^ skeletal muscle,^13^ skin,^14^ periodontal ligament,^15^ deciduous teeth,^16^ and umbilical cord.^17^^,^^18^

Based on the limitations with these BM-MSCs including low number of harvested cells, limited amount of harvested tissues and donor site morbidity or patient discomfort in providing a sample, there was a need for ex vivo expansion or further manipulation of these cells before their preclinical or clinical used to satisfy the safety and efficacy requirements.^19^ Therefore, adipose tissue was considered an attractive alternative source which can be provided in large quantities from adipose tissue fragments .^20^

Based on the reports of the American Society for Aesthetic Plastic Surgery, about five hundred thousand elective liposuction surgeries were undertaken each year in the United States.^21^ So adipose tissue has successfully been used as autologous fat grafts for structural fat grafting in lip, facial, and hand rejuvenation and body contour improvement, but a relatively lower level of attention was directed to their application in cosmetic, plastic, and reconstructive surgery.^22^^-^^26^

Adipose tissue is considered as a source of MSCs, termed adipose-derived stem cells (ASCs). They are ubiquitous and easily obtained in large quantities with little donor site morbidity or patient discomfort^19^ making the use of autologous ASCs an appropriate research tool and cellular therapy.^27^ Lipoaspirates provide an easily obtainable source of ASCs at a frequency of 1:100 to 1:1500 cells exceeding the frequency of MSCs from bone marrow 500-fold, while 1 g of adipose tissue was shown to yield nearly 5,000 ASCs.^28^

ASCs have also the potential for banking as an alternative or complement to cord blood banking for many therapeutic applications in which MSCs could be used.^29^ Several researches were undertaken on tissue remodeling and differentiation of ASCs into specialized somatic cell types to replace damaged organs and tissues.^30^ They were demonstrated to be immunoprivileged^31^ and seem to be more genetically stable in long-term culture^32^ in comparison with BM-MSCs.^33^

Adipose tissue is composed of mature adipocytes constituting about 90% of the tissue volume, and a stromal vascular fraction (SVF) including fibroblasts, endothelial cells, preadipocytes, vascular smooth muscle cells, lymphocytes, resident monocytes/macrophages and ASCs.^34^ ASCs have mesodermal origin, but they have the potential to differentiate into several lineages of osteogenic, chondrogenic, adipogenic, cardiomyogenic, myogenic, and neurogenic cells.^28^ They can differentate into tissues of endo- and ectodermal lineages such as hepatocytes, pancreatic islet cells, endothelial cells, neural cells, and epithelial cells too.^28^

It was shown that ASCs harvested from superficial abdominal regions are significantly more resistant to apoptosis than other parts.^35^ ASCs are available within the brown adipose tissue.^36^ Fresh SVF cells were shown to be heterogeneous with putative ASCs (CD31_, CD34þ/_, CD45_, CD90þ, CD105_, and CD146_), endothelial (progenitor) cells (CD31þ, CD34þ, CD45_, CD90þ, CD105_, and CD146þ), vascular smooth muscle cells or pericytes (CD31_, CD34þ/_, CD45_, CD90þ, CD105_, and CD146þ), and hematopoietic cells (CD45þ) in uncultured conditions..^37^ Also, freshly SVF cells and early passage of ASCs were demonstrated to present higher levels of human leukocyte antigen-DR (HLA-DR), CD117 (c-kit), and stem cell-associated markers such as CD34, along with lower levels of stromal cell markers such as CD13, CD29 (b1 integrin), CD44, CD63, CD73, CD90, CD105, and CD166.^37^ Pericyte markers are also expressed by ASCs such as platelet-derived growth factor (PDGF) receptor-b, smooth muscle b-actin, and neuroglial proteoglycan 2,^38^ while the markers shared by ASCs and MSCs include CD13, CD29, CD44, CD58, and CD166. ASCs like other MSCs can show telomerase activity even lower than the cancer cell lines revealing the capacity of ASCs for self-renewal and proliferation.^39^ Puissant *et al*.^40^ reported the absence of HLA-DR expression and the immunosuppressive properties of ASCs. Fang *et al*. showed that severe steroid-refractory acute graft-versus-host disease (GVHD) could be treated with ASCs from HLA-mismatched donors.^41^

Therefore, cell therapy and regenerative medicine have opened a window as an interdisciplinary field of clinical use of stem cells focussing on the repair, replacement or regeneration of cells, tissue or organs to restore impaired function due to disease, congenital deformities, trauma and ageing. In regenerative medicine, many technological approaches are combined such as stem cell transplantation, gene therapy, the use of soluble molecules, tissue engineering and the reprogramming of cell and tissue types.^42^ Surgeons have tried to market ‘‘stem cell face-lifts’’ when in fact the procedure involving merely facial fat injections.^43^

In regenerative medicine, tissue engineering combines stem cells, growth factors, and biomaterials for repair of failing organs using fabricate biocompatible scaffolds which would promote cell infiltration and angiogenesis together with production of highly purified, bioactive cytokines in large quantity.^44^

ASC therapy in regenerative laboratories and clinical settings was used in treatment of wound beds with a poor blood supply and for healing of radiation injuries. The safety and efficacy of ASCs in reconstructive medicine were evaluated in many clinical trials. The number of these trials had an increasing trend from a total of nine in December 2009 to 18 by May 2010. These trials investigated the efficacy of ASCs in treatment of many diseases and disorders (http://clinicaltrials.gov).

 ASCs were also evaluated in clinical case studies for soft tissue augmentation,^45^ bone tissue repair,^46^ graft-versus-host disease,^47^ immunosuppression^48^ and multiple sclerosis.^49^ Augmentation of soft-tissue deformities using scaffolds seeded with ASCs were previously reported.^50^ ASCs or preadipocytes were used as seeded ones on hyaluronic acid-based scaffolds in 12 volunteers resulting into matrix deposition and cell infiltration. But the hyaluronic acid-based scaffolds could not support pre-adipocyte survival and were not inductive towards adipose tissue formation.^50^

Yoshimura *et al*. used adipose-derived SVF cells in augmentation of soft tissues by cell-assisted lipotransfer (CAL)^51^ in treatment of breast augmentation and facial lipoatrophy. In facial lipoatrophy, no complications or adverse side effects were noticed. 

In augmentation of the breast, the augmentation of breast was successful and had satisfactory clinical results without any major complications too.

In regenerative medicine, skin is an attractive model organ for the use of stem cells. Recent breakthroughs in understanding the role of ASCs in wound healing and tissue regeneration have lead to new options for treating difficult wounds. It was shown in a diabetic animal that the topical administration of autologous ASCs together with a type I collagen sponge matrix could accelerate the healing of diabetic ulcers.^52^

Another clinical study on treatment of radiation-induced tissue damages by administration of human ASCs resulted into progressive improvement in tissue hydration and new vessel formation due to the release of growth factors such as VEGF and HGF leading to the subsequent angiogenesis and proliferation of keratinocytes or dermal fibroblasts.^53^

It was shown that epidermal stem cells might provide a prominent source of multipotent stem cells to replace damaged tissues and lead to wound healing^54^ including a basal keratinocyte population found in the interfollicular epithelium and stem cells residing in the bulge region of the hair follicle.^55^ The other reservoir of skin stem cells is the bulge region of the ORS of hair follicles. Stem cells provided from hair follicles could be differentiated into the ORS of the hair follicle.^56^

The application of ASCs and other stem cells in treatment of many medical conditions has been reflected in several mass media. But it is of great importance in scientific and medical communities to balance the hope from the hype and just time will clarify whether, someday, healthy subjects will voluntarily undergo liposuction to donate fat identically when they donate blood.^56^^,^^57^ Also as ASCs have gained popularity in regenerative medicine, improvement in methods to assess their reproducibility, safety and quality of the vitro expanded stem cells is important. In addition, producing cells that are genetically stable is a step towards getting insurance that the cells would not transform, leading to genetically aberrated progeny when transplanted into the recipient.^57^

Based on the available data in literature, further studies are needed in both basic and clinical sciences on application of ASCs to clarify if ASC-supplemented fat transplants are really safe and effective and also on the costs, graft survival, predictability of findings, ease of procedures and aesthetic outcomes. 

## CONFLICT OF INTEREST

The authors declare no conflict of interest.

## Footenote

Please cite this paper as:

Mehrabani D, Mehrabani G, Zare Sh, Manafi A. Adipose-Derived Stem Cells (ADSC) and Aesthetic Medicine: A Mini Review. World J Plast Surg 2013;2(2): 65-70.
